# Immunofluoresence of the Outer Root Sheath in Anagen and Telogen Hair: An Aid to Diagnosis in Pemphigus

**DOI:** 10.4103/0974-7753.58558

**Published:** 2009

**Authors:** M Kumaresan, Reena Rai, V Sandhya

**Affiliations:** Department of Dermatology, PSG Hospitals, Coimbatore, India; 1 Department of Pathology, PSG Hospitals, Coimbatore, India

**Keywords:** Immunofluoresence, outer root sheath, telogen hair

## Abstract

Desmoglein 1 and 3 are distributed in the outer root sheath (ORS) of the hair follicle. Direct immunofluoresence (DIF) pattern of ORS in cases of pemphigus resembles the DIF pattern of the perilesional skin. We performed a DIF of the anagen and telogen hair ORS in a case of pemphigus and correlated it with the DIF findings of perilesional skin. Telogen hair ORS promises to be a useful tool in performing DIF for the purpose of diagnosis and follow-up in cases of pemphigus

## INTRODUCTION

Pemphigus is a group of autoimmune blistering disease of the skin and mucosa characterized by antibodies against desmoglein (Dsg) 3 and Dsg 1. The outer root sheath (ORS) of the hair follicle is structurally analogous to epidermal keratinocytes.[1] Pemphigus antigens are distributed throughout the ORS and in the dermal bulb matrix cells.[1] Pemphigus-specific immunofluorescence pattern seen in the skin has been demonstrated in the ORS of a plucked hair follicle.[2-4] Dsg 3 is responsible for anchoring the telogen hair to the follicle.[5] The increase in volume of the target antigen (Dsg 3 and Dsg 1) in the follicular epithelium could be a factor in determining scalp involvement in pemphigus. We performed direct immunofluoresence (DIF) of the anagen and telogen hair ORS in a case of pemphigus vulgaris and correlated it with the DIF findings of perilesional skin.

## CASE REPORT

**Figure 1:**
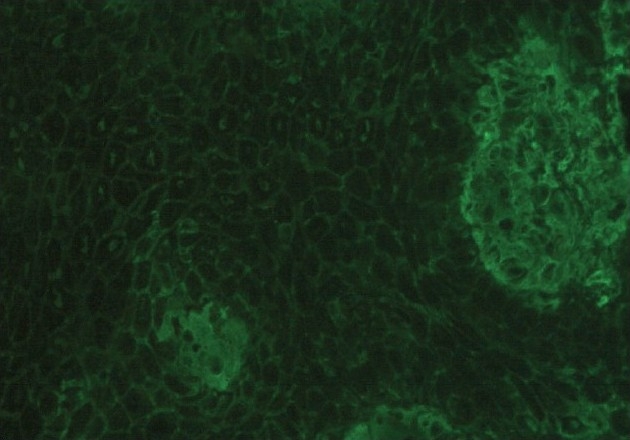
Perilesional skin direct immunofluoresence showing intercellular depostis of immunoglobulin G

A 36-year-old male presented with multiple flaccid blisters and oozy erosions over the scalp, trunk, and oral cavity of 20 days duration. There was no history of similar complaints in the past. On examination, he had severe erosions with oozing over the scalp and patchy hair loss. Flaccid blisters were seen on the trunk and upper limbs. The oral cavity showed erosins. Nikolsky's sign was positive. With the above findings, a clinical diagnosis of pemphigus vulgaris was made. Skin biopsy revealed features consistent with pemphigus vulgaris. DIF of perilesional skin revealed the typical fish net pattern of intercellular immunoglobulin (Ig) G and C3 deposits [Figure 1]. Telogen hair and anagen hairs were selected by the hair pull test and identification under microscope. Hair samples were subjected to DIF without sectioning by directly mounting it on the slide. The ORS of both anagen and telogen hair showed the characteristic fish net or chicken wire pattern seen in pemphigus [Figure 2a and b].

**Figure 2a:**
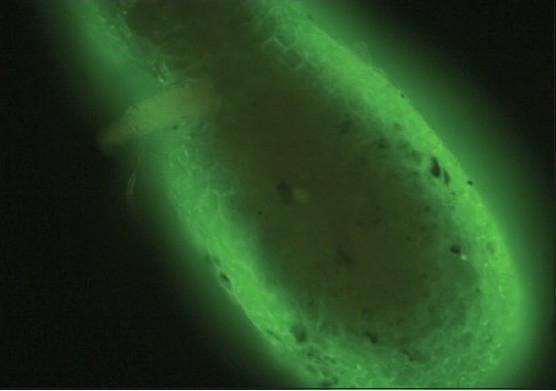
Direct immunofluoresence pattern of the outer root sheath in telogen hair

**Figure 2b:**
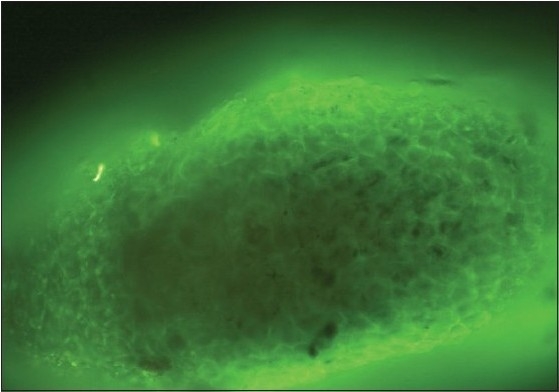
Direct immunofluoresence pattern of the outer root sheath in anagen hair

## DISCUSSION

Pemphigus is a group of autoimmune blistering disease of the skin and mucosa characterized by antibodies against Dsg 3 and, to a lesser extent, against Dsg 1. DIF of perilesional skin and mucosa has been established as the confirmatory test for diagnosis. DIF in pemphigus shows IgG antibodies deposited in a fish net or chicken wire pattern against Dsg 1 and Dsg 3. The cell adhesion proteins of the desmosome, Dsg and desmocollin are members of the cadherin family of cell adhesion molecules. They are transmembrane proteins that bridge the space between adjacent epithelial cells by way of homophilic binding of their extracellular domains to other desmosomal cadherins on the adjacent cell. Within stratified squamous epithelia, such as the epidermis and ORS, Dsg are generally expressed in a differentiation-zspecific manner.[6] Dsg 1 is expressed in epidermal suprabasal cells, the inner root sheath, and the innermost layers of the ORS. Dsg 3 is expressed throughout the ORS. In areas of epidermal-like keratinization, such as in the infundibulum, Dsg 3 expression is mainly limited to the basal layer.[6] Dsg 3 is responsible for anchoring the telogen hair to the follicle.[5]

Immunodeposits specific to pemphigus were previously shown in the ORS and matrix of hair follicle biopsy specimens.[7] Schaerer and Trueb demonstrated the practicability of DIF on plucked hair for the first time.[2] They were able to demonstrate the pemphigus-specific DIF pattern in 100% of their cases. Ragavendra *et al*.[3] demonstrated an 85% DIF positivity in ORS of plucked anagen hair. Daneshpazhooh *et al*.[4] reported a 91% DIF positivity in the ORS of plucked hair among 110 cases of pemphigus vulgaris.

In our case, pemphigus-specific DIF pattern was seen in the ORS of telogen hair, which correlated with the DIF findings of anagen and perilesional skin. DIF positivity in the ORS of telogen hair has not been reported. Because telogen hair is rich in Dsg 3, it may act as a good substrate for performing DIF in cases of pemphigus. Telogen hair is readily obtained by gentle pulling or combing the hair and is less painful than plucking. Telogen hair ORS promises to be a useful tool in performing DIF for the purpose of diagnosis and follow-up in cases of pemphigus. For processing the hair as a substrate for DIF, we mounted the hair directly on the slide without sectioning. This method may overcome the technical difficulties encountered during the earlier studies.[4] However, because ours is a single case report, we need a large sample size to validate this method.
